# Ym155 localizes to the mitochondria leading to mitochondria dysfunction and activation of AMPK that inhibits BMP signaling in lung cancer cells

**DOI:** 10.1038/s41598-022-17446-y

**Published:** 2022-07-30

**Authors:** Arindam Mondal, Dongxuan Jia, Vrushank Bhatt, Moumen Akel, Jacques Roberge, Jessie Yanxiang Guo, John Langenfeld

**Affiliations:** 1 Department of Surgery, Rutgers Robert Wood Johnson Medical School, Rutgers, The State University of New Jersey, New Brunswick, NJ 08903 USA; 2 Rutgers Cancer Institute of New Jersey, New Brunswick, NJ 08901 USA; 3 Rutgers University, Piscataway, NJ 08854 USA; 4 Molecular Design and Synthesis, RUBRIC, Office for Research, Rutgers Translational Science, Rutgers University, Piscataway, NJ 08854 USA

**Keywords:** Cancer, Cell biology

## Abstract

The imidazolium compound Ym155 was first reported to be a survivin inhibitor. Ym155 potently induces cell death of many types of cancer cells in preclinical studies. However, in phase II clinical trials Ym155 failed to demonstrate a significant benefit. Studies have suggested that the cytotoxic effects of Ym155 in cancer cells are not mediated by the inhibition of survivin. Understanding the mechanism by which Ym155 induces cell death would provide important insight how to improve its efficacy as a cancer therapeutic. We demonstrate a novel mechanism by which Ym155 induces cell death by localizing to the mitochondria causing mitochondrial dysfunction. Our studies suggest that Ym155 binds mitochondrial DNA leading to a decrease in oxidative phosphorylation, decrease in TCA cycle intermediates, and an increase in mitochondrial permeability. Furthermore, we show that mitochondrial stress induced by Ym155 and other mitochondrial inhibitors activates AMP-activated kinase leading to the downregulation to bone morphogenetic protein (BMP) signaling. We provide first evidence that Ym155 initiates cell death by disrupting mitochondrial function.

## Introduction

Ym155 was originally reported to be a small molecule inhibitor of survivin^1^. Survivin belongs to the family of anti-apoptotic proteins^2^. Ym155 has been shown to potently inhibit growth at 10–20 nM concentrations in a vast number of cancer cell lines including non-small lung cancer, renal, glioblastomas, triple negative breast cancer, prostate, AML, and others^1,3–7^. Ym155 has had only modest benefits in phase I/II trails but was well tolerated^8,9^. The mechanism(s) by which Ym155 induces such potent cell death of cancer cells in vitro is poorly understood.

Many of the growth suppressive effects of Ym155 cannot be attributed to the suppression of survivin^10^. Ym155 induces a DNA damage response that is not mediated by the inhibition of survivin^10–12^. In a model in which BCL-xl is suppressed by siRNA, Ym155 caused significantly more apoptosis and increase in mitochondrial permeability compared to other therapeutics^13^. However, the role of survivin inhibition causing an increase in mitochondrial permeability was not established^13^. Ym155 was recently reported to active AMP-activated kinase (AMPK), which inhibits mTOR signaling^14^. The mechanism by which Ym155 activates AMPK has not been elucidated.

Ym155 was recently reported to potently inhibit bone morphogenetic protein (BMP) signaling in lung cancer cells that was not mediated by the inhibition of survivin^15^. The mechanism by which Ym155 decreases BMP signaling is not known. Recently, AMPK was shown to suppress BMP signaling in lung cancer cells and *C elegans*^16,17^. The combination of BMP small molecule inhibitors with Ym155 synergistically induced caspase-independent cell death that involved apoptosis inducing factor (AIF) being released from the mitochondria and localizing to chromosomal DNA. AIF nuclear localization was dependent on the “hyperactivation” of AMPK. These studies led us to the question whether Ym155 was targeting the mitochondria.

In the present study, we show that Ym155 localizes to the mitochondrial DNA (mtDNA) of lung cancer cells causing a rapid decline in oxidative phosphorylation and decrease in TCA intermediates. Ym155 and other cancer therapeutics targeting the mitochondria activate AMPK and decrease BMP signaling. These studies demonstrate novel mechanisms by which Ym155 targets the mitochondria to promote death of cancer cells.

## Results

### Structurally Ym155 is similar to Ethidium Bromide

**Figure 1:**
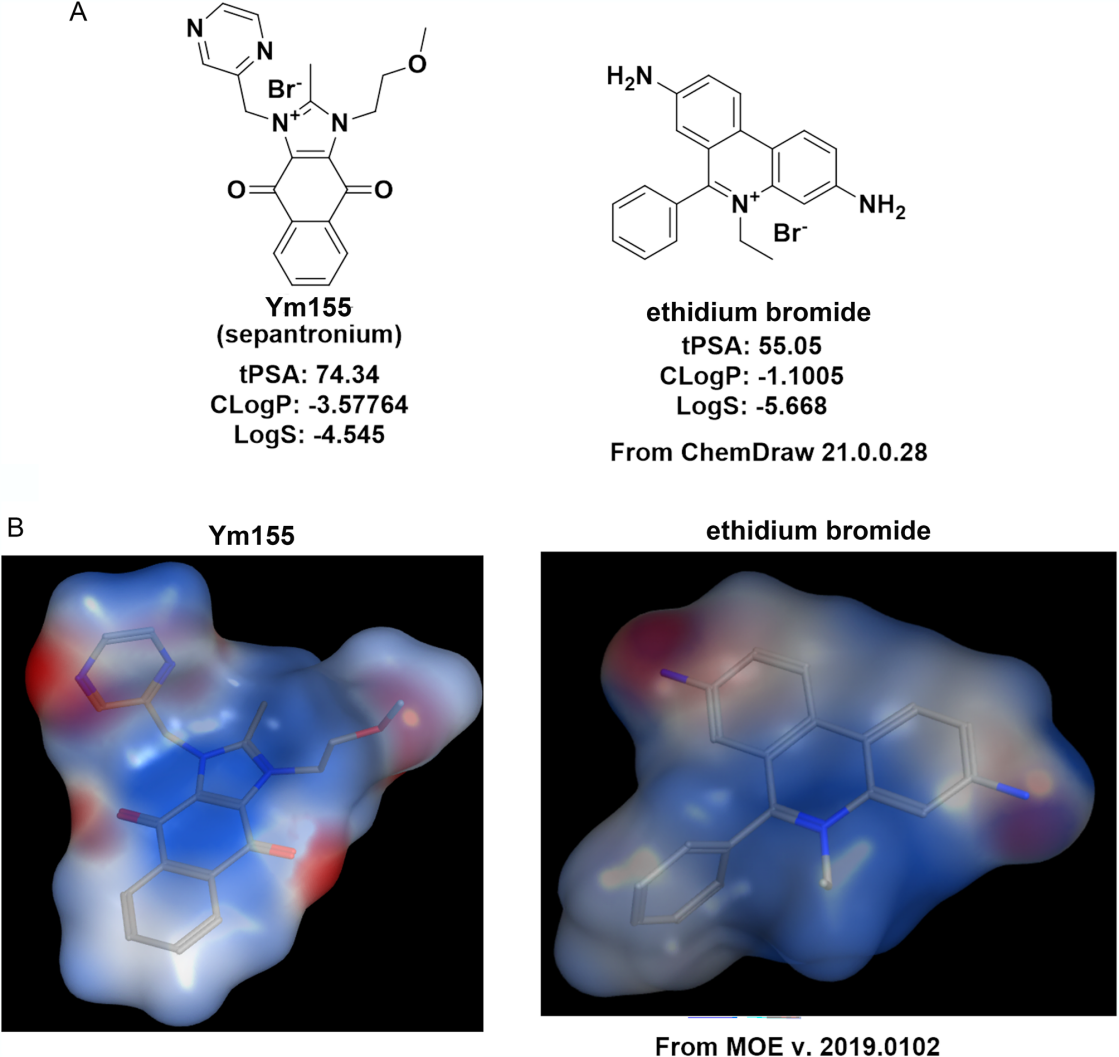
Structural properties of Ym155 are similar to Ethidium Bromide: (**A**) Structures of Ym155 and ethidium bromide with calculated topological polar surface area (tPSA), octanol–water partition coefficient (CLogP), and LogS. (**B**) Similarities of the electrostatic charge distributions of Ym155 and ethidium bromide determined with Molecular Operating Environment (MOE) software.

To better understand the mechanism of action of Ym155, we profiled its physical properties to known compounds. Ym155 shares structural similarities and charge distribution with ethidium bromide (Fig. 1A). The topological polar surface area (tPSA), octanol–water partition coefficient (CLogP), and LogS of Ym155 are similar to ethidium bromide. The electrostatic charge distribution of Ym155 is also similar to ethidium bromide (Molecular Operating Environment, MOE) (Fig. 1B). Because of the high membrane potential, mitochondria are the most negatively charged organelle, which attracts the positively charged compounds^18,19^. Like many conjugated lipophilic cations, ethidium bromide targets the mitochondria and mtDNA due to its charge distribution . Because of the similarities to ethidium bromide, Ym155 was predicted to target the mitochondria and mtDNA.

### Ym155 localizes to mitochondrial DNA

**Figure 2:**
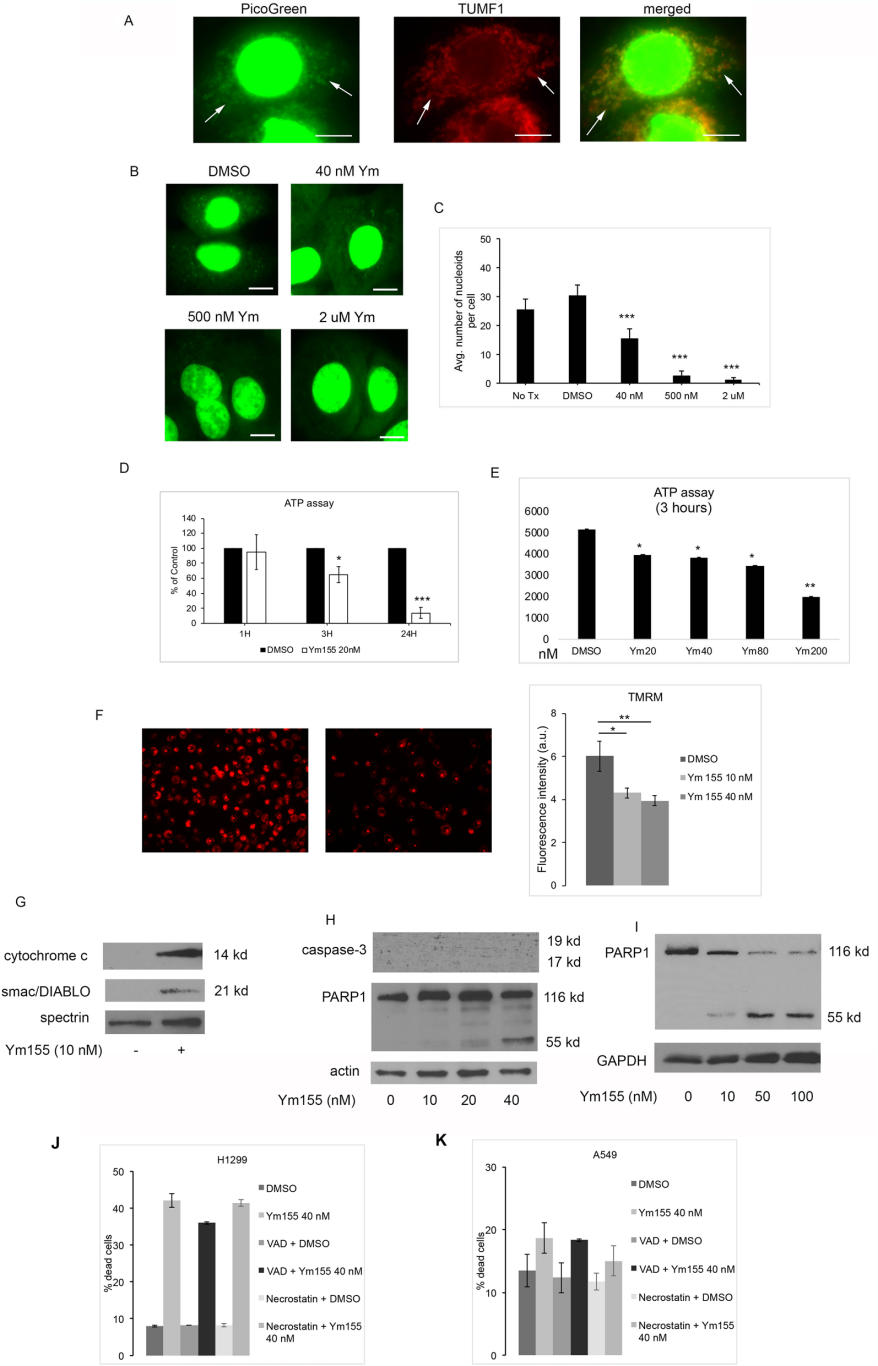
Ym155 binds mitochondrial DNA and disrupts mitochondrial function. (**A**) Dual immunofluorescent imaging of PicoGreen and mitochondrial marker TUMF in A549 cells. PicoGreen stained mitochondria are in the cytoplasm (arrows). Mitochondria staining both PicoGreen and TUMF are orange in merged images. (**B**) Representative images of PicoGreen mitochondrial staining with increasing doses of Ym155. (**C**) Quantification of PicoGreen cytoplasm fluorescence demonstrating Ym155 quenches PicoGreen staining. (**D**) ATP concentration of H1299 cells treated with Ym155 20 nM up to 24 h, presented as percent of control (*n* = 4). (E) Mean of 2 experiments for ATP concentration of H1299 treated increasing concentrations of Ym155 for 3 h. (**F**) TMRM assay of mitochondrial membrane potential and fluorescence quantification of H1299 cells treated with Ym155 for 3 h (20 × magnification). (**G**) Immunoblot of cytosol of H1299 cells treated with Ym155 for 24 h. To include results for only Ym155 treated cells the image was cropped (Supplementary Information). (**H**) Immunoblot for activated caspase-3 and PARP expression of A549 cell treated with Ym155 for 24 h. (**I**) Immunoblot of H1299 cells treated with Ym155 for 48 h. (**J**–**K**) Mean cell death of two experiments of H1299 and A549 cells treated with Ym155 for 24 h. with and without 50 μM Z-VAD-FMK (VAD) or 20 μM necrostatin. *p < 0.05, **p < 0.01, ***p < 0.001.

DNA binding agents are widely used as chemotherapeutic agents. DNA binding drugs predominately accumulate in chromosomal DNA, however, some exert their anti-cancer effects by intercalating mitochondrial DNA (mtDNA)^20^. Chemotherapeutic agents binding with mtDNA have been shown to interfere with mitochondrial function leading to a depletion of ATP^21^. Quenching the fluorescent signaling of DNA binding agents with propidium bromide and Hoescht 33,342 have been used to monitor accumulation of DNA binding drugs. PicoGreen is a fluorescent probe, which selectively labels both nuclear and mtDNA and is used to monitor the accumulation of drugs in mtDNA^20,22^. We utilized PicoGreen quenching studies to assess if Ym155 localizes to the mtDNA. PicoGreen fluorescent signaling was found in the nucleus and cytoplasm of A549 cells (Fig. 2A). Dual immunfluorescent imaging of PicoGreen with the mitochondrial marker TUMF showed co-localization of PicoGreen with TUMF, demonstrating PicoGreen localizes to the mitochondria (Fig. 2A). Increasing concentrations of Ym155 showed a dose-responsive quenching of PicoGreen fluorescence in the mitochondria (Fig. 2B, C).

To assess whether Ym155 affects mitochondrial function, we examined whether ATP was depleted in H1299 cells following treatment with Ym155. Low dose of Ym155 (20 nM) caused a small decrease in ATP levels at 3 h, which after 24 h was substantially lower than DMSO (Fig. 2D). Ym155 caused a dose-related decrease in ATP levels as early as 3 h in H1299 cells (Fig. 2E).

### Ym155 decreases mitochondrial membrane potential and increases mitochondrial permeability

TMRM accumulates in negatively charged polarized mitochondrial, which fluoresces red. When mitochondrial membrane potential collapses TMRM is dispersed into the cytosol decreasing fluorescence levels. Ym155 caused a dose-related decrease mitochondrial membrane potential within 3 h (Fig. 2F). Ym155 caused an increase in mitochondrial proteins cytochrome c and smac/DIABLO expression in the cytosol, indicating increased mitochondrial permeabilization (Fig. 2G). Cytochrome c released into the cytosol activates caspases and smac/DIABLO is an inhibitor of the anti-apoptotic proteins, both of which can induce apoptosis.

### Ym155 does not activate apoptosis

During apoptosis, procaspase-3 is cleaved into active 17 and 19 kd fragments. Activated caspase-3 cleaves poly (ADP-ribose) polymerase-1 (PARP1) to 85 kd fragment. Ym155 did not activate caspase-3 in either A549 or H1299 cells (Fig. 2H and data not shown). The predominant cleaved PARP fragment was 55 kd (Fig. 2H, I). The 55 kd fragment was more evident with increasing exposure to Ym155 (Fig. 2I). These data suggest PARP1 cleavage was from proteases other than caspases^23^. We recently reported that Ym155 in combination with the BMP inhibitor JL5, there was increased lysosome permeability with cathepsins released into the cytoplasm promoting cell death^15^. In addition, the caspase inhibitor Z-VAD-FMK and the necrosis inhibitor necrostatin did not inhibit cell death induced by Ym155 (Fig. 2J,K). These data suggest that Ym155 induced cell death is independent of apoptosis and necrosis.

### Ym155 decreases oxidative phosphorylation

**Figure 3:**
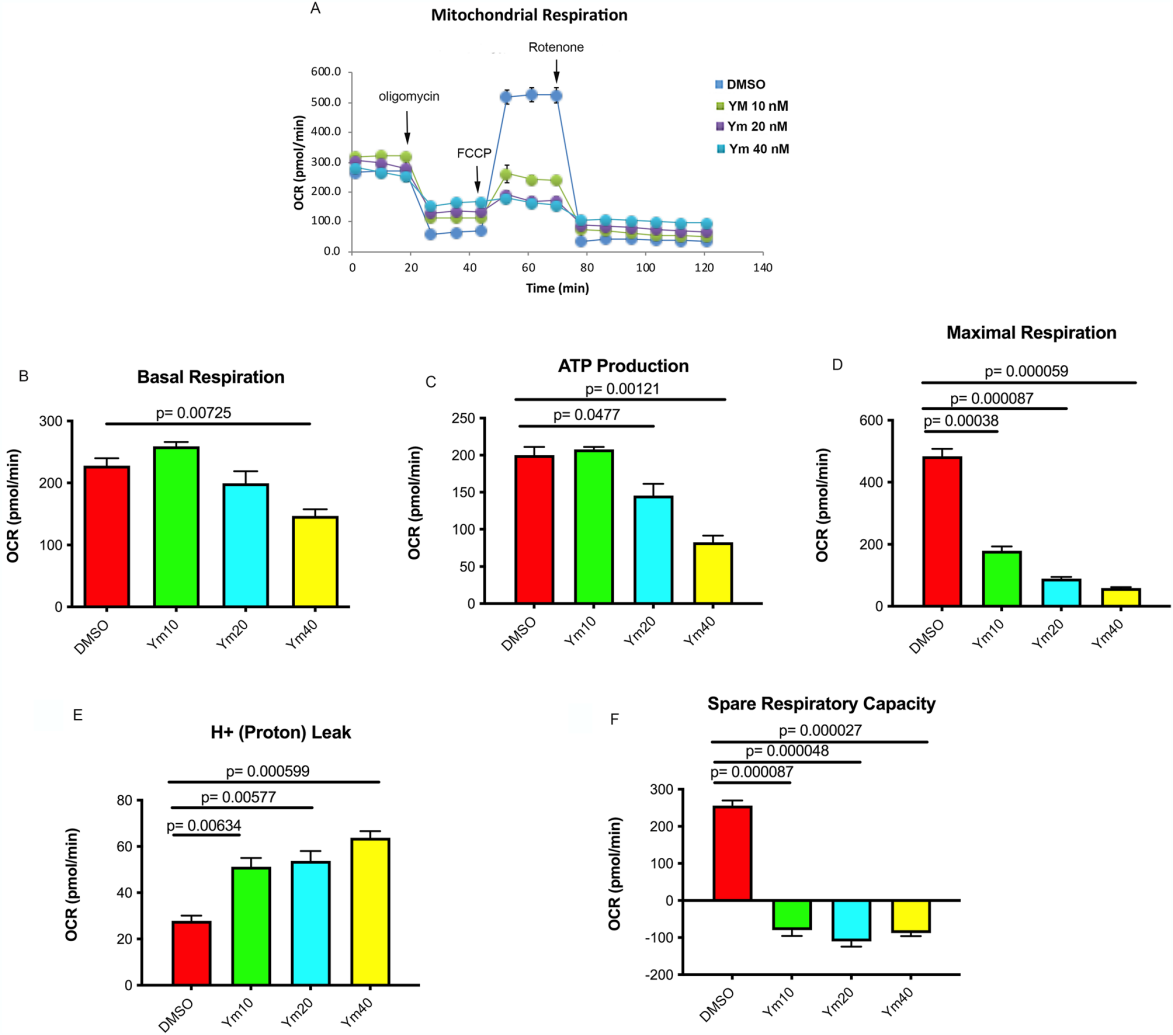
Ym155 decreases mitochondrial respiration: The Seahorse XFe24 analyzer was used to monitor oxygen consumption rate (OCR) and examine oxidative phosphorylation of H1299 cells treated with Ym155 for 3 h. Inhibitors of ETC oligomycin (complex V), FCCP (uncoupler), Rotenone (complex I) were used to interrogate oxidative phosphorylation. (**A**) OCR curves of H1299 cells treated with Ym155. (**B**) Mean basal respiration, (**C**) ATP production, (**D**) maximal respiration, (**E**) proton leak, and (**F**) spare capacity from 3 experiments.

The Seahorse analyzer (eXF24) was used to determine the effects of Ym155 on oxidative phosphorylation. Changes in oxygen consumtion following treatment with mitochondrial inhibitors are shown in Fig. 3A. Within 2 h, Ym155 decreased basal respiration of H1299 cells (Fig. 3B). Oligomycin blocks ATP synthase inhibiting ATP production from ADP. Oligomycin treatment indicated that Ym155 decreased ATP production (Fig. 3C). FCCP depletes the proton gradient, uncoupling respiration from ATP synthesis allowing for determination of maximum respiration. Ym155 significantly suppressed maximum respiration (Fig. 3D), increased proton leak (Fig. 3E), and decreased spare respiratory capacity (Fig. 3F). These studies reveal that Ym155 significantly inhibits oxidative phosphorylation in lung cancer cells supporting the view that Ym155 targets the mitochondria.

### Ym155 alters the levels of intermediates involved in glycolysis, TCA cycle and pyrimidine metabolism

**Figure 4:**
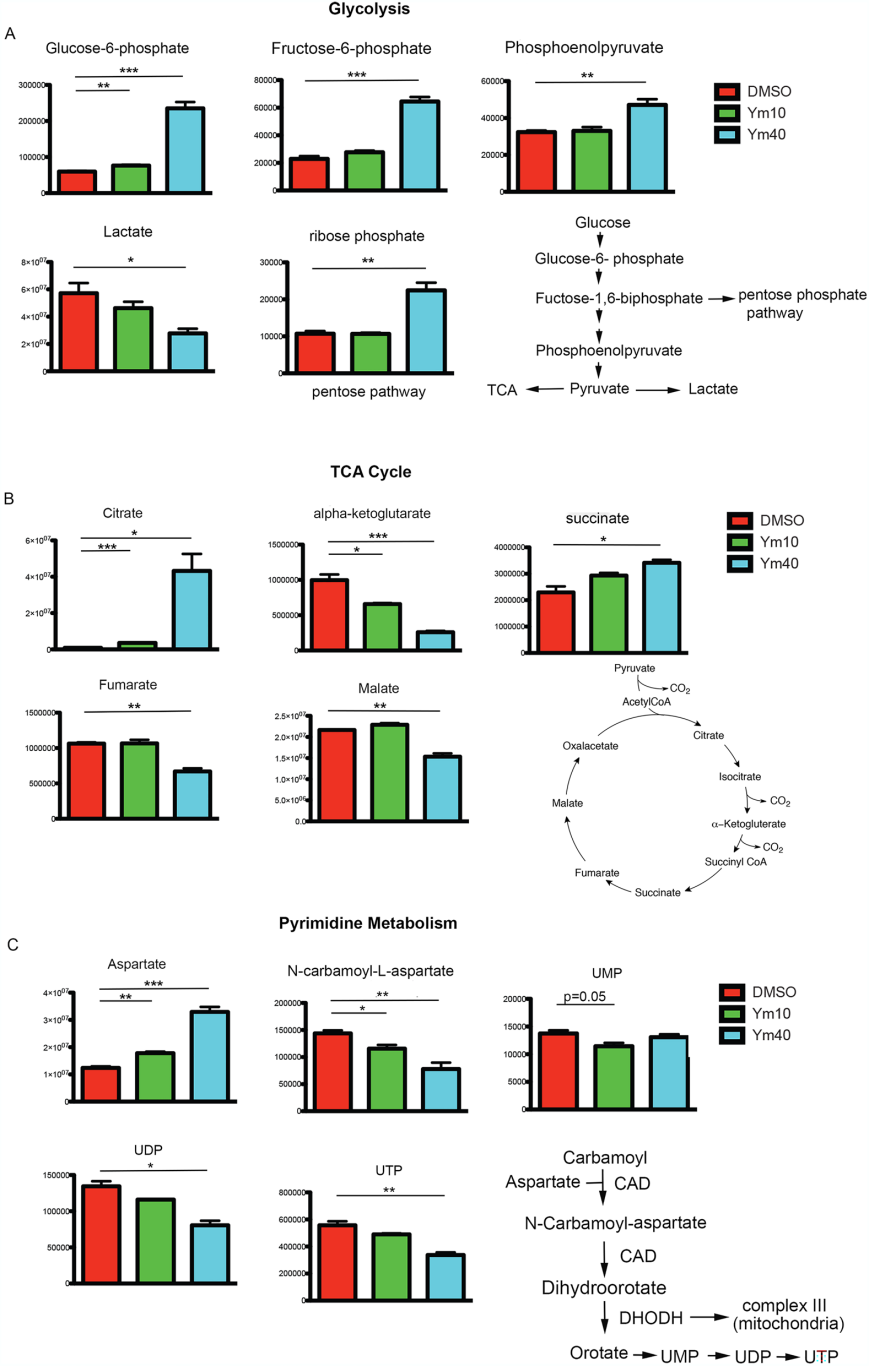
Ym155 decreases TCA cycle metabolite intermediates. Metabolite intermediates of glycolysis, TCA cycle, and pyrimidine intermediates were determined by MS/LS/MS of H1299 cells treated with Ym155 for 3 h. (**A**) Early and late glycolysis intermediates were increased with Ym155 as wells as the pentose pathway intermediate ribose phosphate. (**B**) TCA intermediates alpha-ketoglutarate, fumarate, and malate were decreased. Citrate and succinate metabolites were increased. (**C**) Pyrimidine intermediates were decreased following Ym155 treatment. CAD (carbamoyl aspartate dehydrogenase), DHODH (dihydroorotate dehydrogenase). *p < 0.05, **p < 0.01, ***p < 0.001.

To further elucidate how Ym155 affect cancer cell metabolism, metabolomics profiling of H1299 cells treated with Ym155 for 3 h were analyzed using LC–MS. The level of glycolytic intermediates glucose-6-phosphate, fructose-6-phosphate and phosphoenolpyruvate were significantly increased in H1299 cells treated with Ym155 compared with vehicle control (Fig. 4A). The level of pentose phosphate intermediate, ribose phosphate, was also increased when cells were treated with Ym155 (Fig. 4A). However, the levels of lactate and most tricarboxylic acid (TCA) cycle intermediates, including α-KG, fumarate and malate were decreased by Ym155 treatment; while the levels of citrate and succinate were increased in cells treated with Ym155 (Fig. 4B).

Complex II of the ETC (electron transport chain) catalyzes the oxidation of succinate to fumarate^24^. Complex II is essential for the generation of TCA intermediates. These studies suggest that the decrease in TCA cycle intermediates induced by Ym155 could be caused by inhibition of the ETC.

Mitochondrial electron transport is coupled with pyrimidine metabolism by dihydroorotate dehydrogenase (DHODH). DHODH catalyzes the oxidation of dihydrooratate to orotate, which requires transfer of electrons from dihydrooratate to ubiquinone of mitochondria complex III^25^. DHODH activity and subsequent pyrimidine metabolism is dependent on complex III function^25^. We found that intermediates of pyrimidine metabolism including N-carbomyl-L-aspartate, UDP, and UTP were also decreased in H1299 cells treated with Ym155 for 3 h (Fig. 4C).

### Ym155 inhibited glucose carbon flux to TCA cycle intermediates

**Figure 5:**
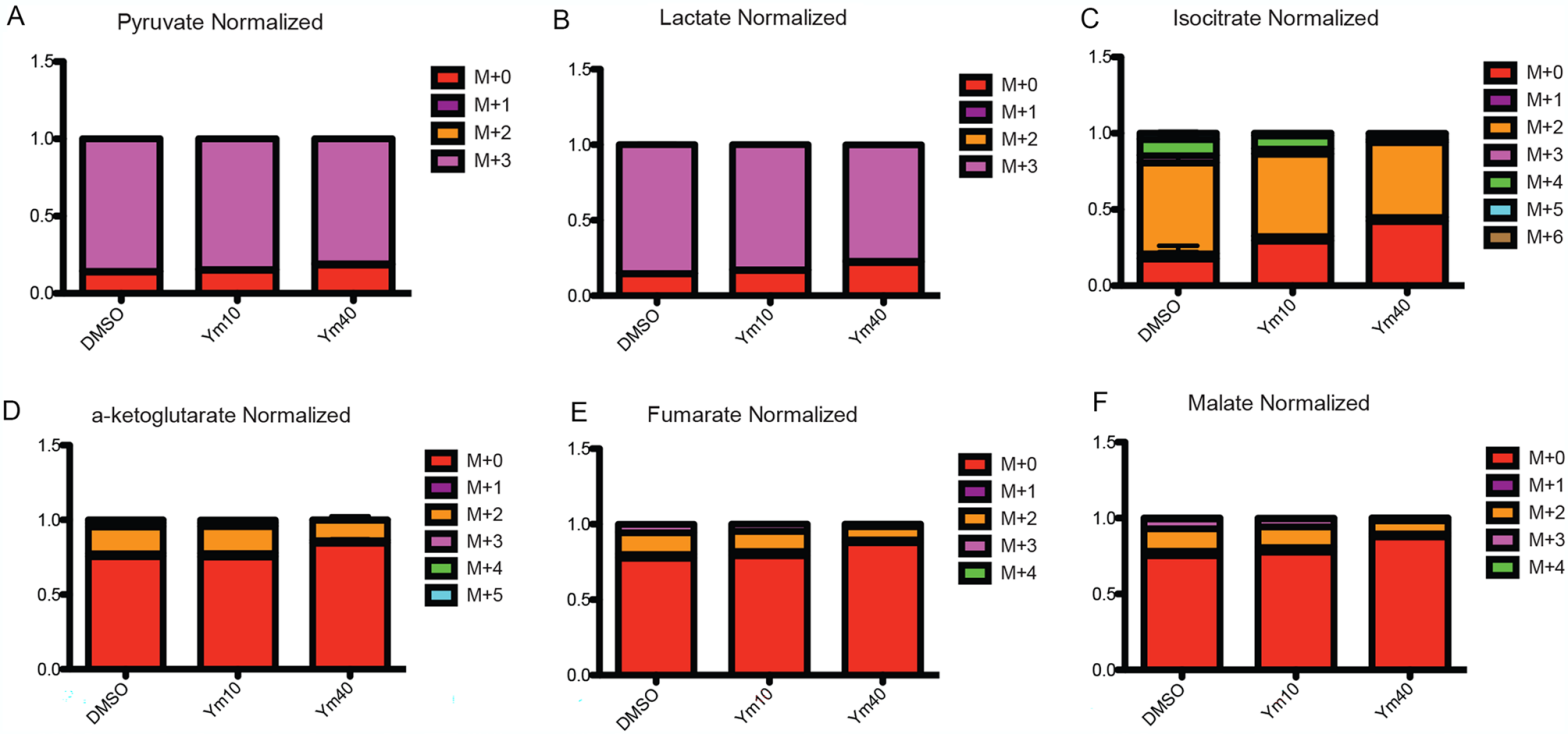
Ym155 decreases glucose 2-carbon transfer in the TCA cycle. H1299 cells were loaded with [U13C6] glucose for 2 h then treated with Ym155 for 3 h. (**A**, **B**) The number 3 carbons (M + 3) in glycolysis intermediates pyruvate and lactate was the same between Ym155 treated and control. (**D**) The TCA cycle intermediate isocitrate, alpha-ketoglutarate, fumarate, and malate had significantly lower M + 2 carbons in Ym155 treated cells compared to controls (**C**–**F**).

To further investigate the mechanism by which Ym155 decreases the levels of TCA cycle intermediates, [U-13C6] glucose in vitro tracing followed by LC/MS and metabolic flux analysis was performed in H1299 cells treated with vehicle control or Ym155 at increasing concentrations for 3 h. Glucose is metabolized through glycolysis as 3 carbon intermediates. Two carbons of pyruvate enter the TCA cycle as acetylCoA to form citrate. YM155 did not alter glucose carbon flux to pyruvate and lactate (Fig. 5A, B). This is consistent with the metabolomic studies demonstrating that Ym155 does not inhibit glycolysis. However, tracing the 2 carbons of glucose through the TCA cycle showed a decrease in isocitrate, alpha-ketoglutarate, fumarate and malate in Ym155 treated cells compared to controls (Fig. 5C–F), indicating that Ym155 impairs mitochondrial metabolism, which may be mediated by the inhibition of the ETC.

### Ym155 activates AMPK

**Figure 6:**
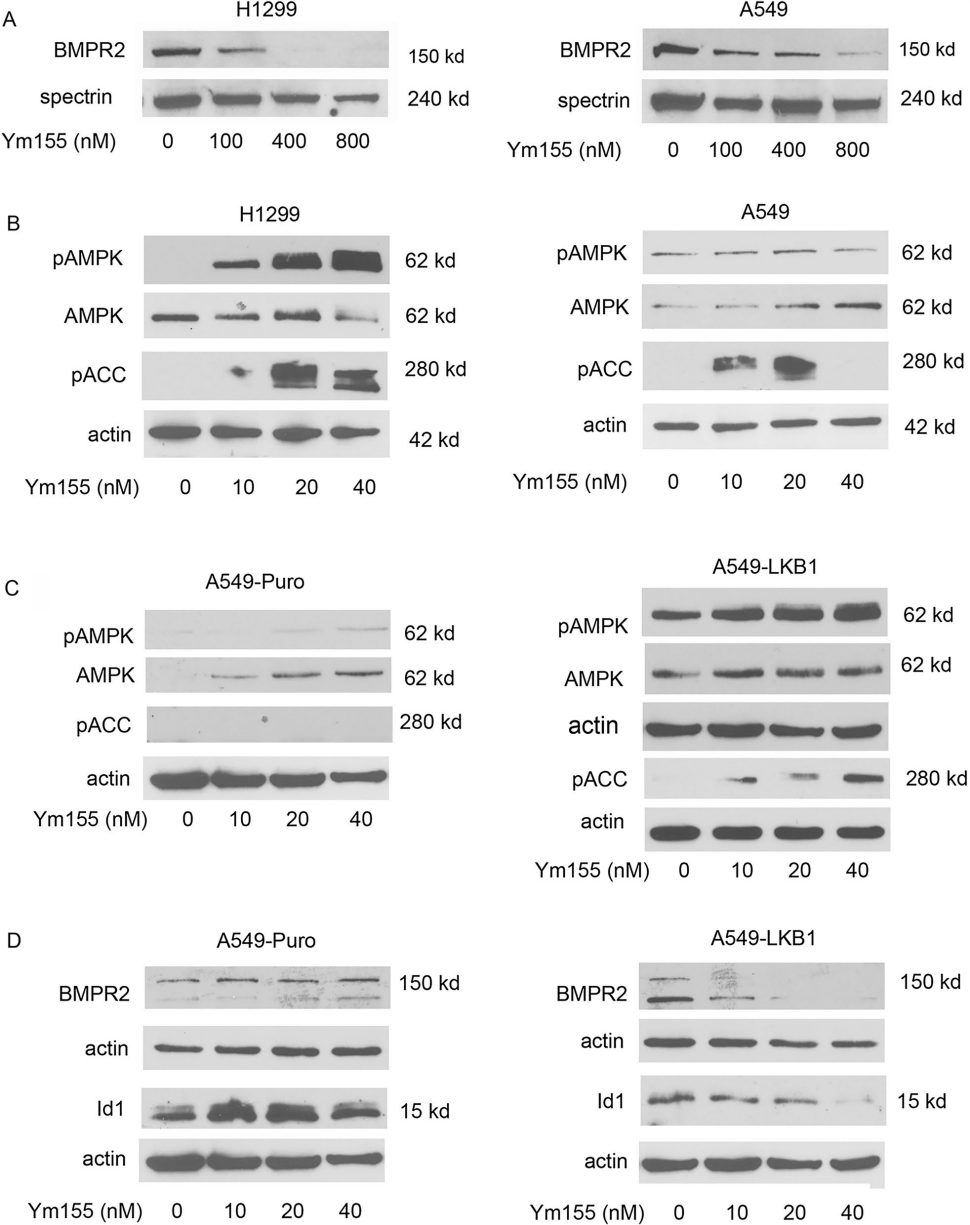
Ym155 activates AMPK and downregulates BMP signaling. (**A**) Immunoblot for BMPR2 in H1299 and A549 cells treated with Ym155 for 24 h. (**B**) Immunoblot for pAMPK (T172) and pACC (Ser79) of cells treated for 24 h. (**C**) Immunoblot for pAMPK (T172) and pACC (Ser79) of A549 control transfected cells (A549-puro) and A549 cells stably transfected with LKB1 (A549-LKB1) treated for 24 h. (**D**) Immunoblot of A549-puro and A549-LKB1 cells treated with Ym155 for 24 h.

We previously reported that Ym155 potently decrease Id1 and pSmad-1/5 expression of the BMP signaling cascade in H1299 and A549 lung cancer cell lines^15^. We recently reported that AMPK decreases BMP signaling in lung cancer cells and in *C elegans*^17,26^. Since Ym155 decreases mitochondrial function and ATP levels, we asked if Ym155 activated AMPK effecting BMP signaling. Ym155 decreased the expression of BMPR2 in both H1299 and A549 cells (Fig. 6A). AMPK is activated by cellular stress and a decrease in ATP production, which occurs with the inhibition of oxidative phosphorylation. A549 cells lack LKB1, which phosphorylates AMPK α-subunit at T172 leading to activation^27^. Allosteric activation of AMPK can occur without LKB1^27^. Ym155 induced phosphorylation of AMPK at T172 indicating activation in H1299 cells but not in A549 cells (Fig. 6B). In A549 cells, Ym155 induced phosphorylation of acetyl-CoA-carboxylase 1 (ACC1) at Ser79 (Fig. 6B), the downstream target of AMPK^28^, indicating allosteric activation of AMPK. In A549 cells stably expressing LKB1, Ym155 caused a more potent activation of AMP (Fig. 6C). Ym155 caused a greater decrease in Id1 and BMPR2 expression in A549-LKB1 cells compared to A549-puro cells (Fig. 6D). These studies show that Ym155 potently activates AMPK in lung cancer cells, which likely occurs from disrupting mitochondrial function. The data also suggests that Ym155 decreases BMP signaling by activating AMPK.

### Mitochondrial inhibitors activate AMPK and downregulate BMP signaling

**Figure 7:**
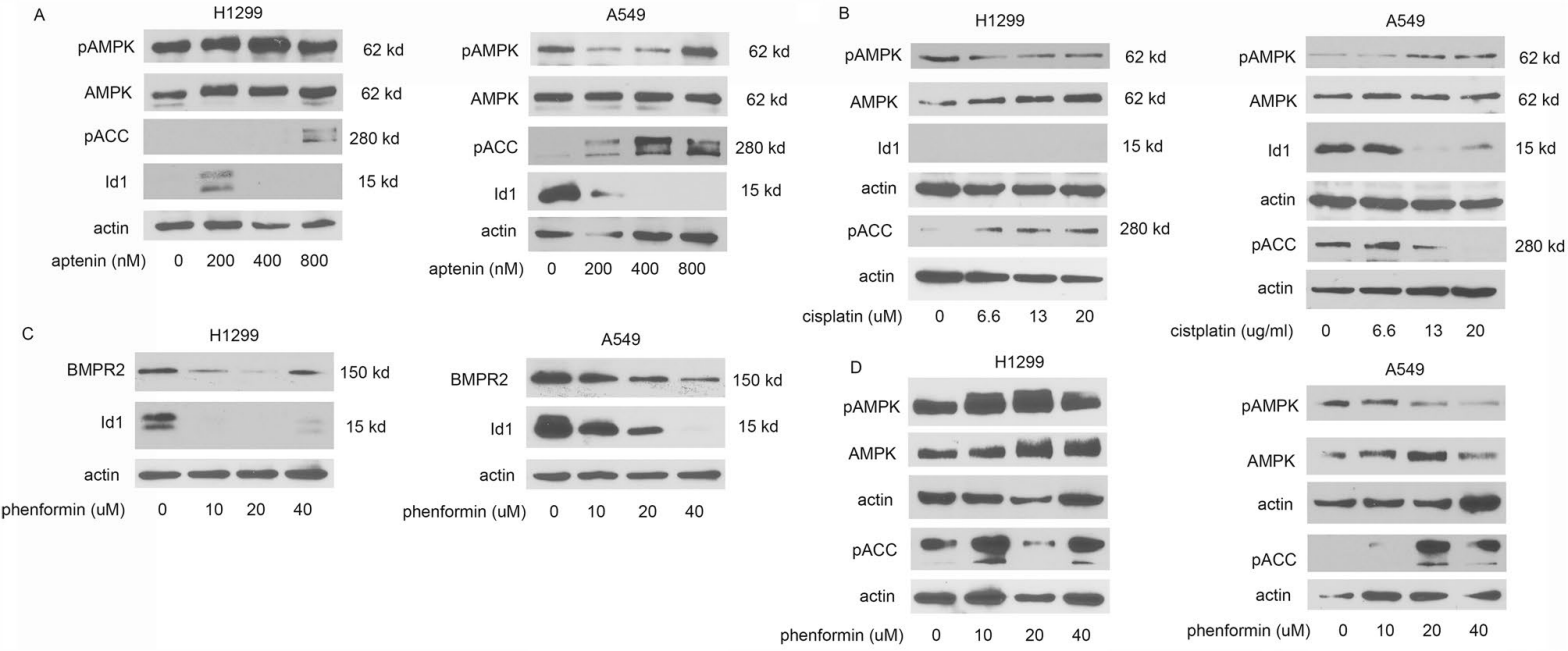
Mitochondrial inhibitors activate AMPK and downregulate BMP signaling. H1299 and A549 cells were treated with inhibitors for 24 h. (**A**) Immunoblots for cells treated with atpenin, (**B**) cisplatin, or (**C**–**D**) phenformin.

Next, we examined if other inhibitors of mitochondrial ETC activate AMPK and downregulate BMP signaling. Atpenin A5 inhibits complex II (succinate-ubiquinone reductase) of the ETC^29^. In H1299 cells, atpenin A5 activates AMPK and pACC and decreases the expression of Id1 (Fig. 7A). In A549 cells, atpenin activated pACC1 and decreased expression of Id1 (Fig. 7A). Cisplatin is a DNA intercalating chemotherapeutic agent is thought to mediate its cytotoxic effects by localizing to the mitochondria^30^. Cisplatin did not activate pAMPK in either cell lines but did activate ACC1 (Fig. 7B). Cisplatin also significantly decreased Id1 expression in A549 cells (Fig. 7B). Id1 expression was not identified in H1299 in these experiments (Fig. 6B). Phenformin inhibits complex I of the electron transport chain, decreasing oxidative phosphorylation, and activates AMPK^31^. In both H1299 cells and A549 cells, phenformin decreased expression of BMPR2 and Id1 (Fig. 7C). Phenformin activated AMPK in H1299 and ACC1 in both H1299 and A549 cells (Fig. 7D). These studies demonstrate that therapeutics targeting mitochondrial ETC and activate AMPK cause a decrease BMP signaling, supporting the view that Ym155 decreases BMP signaling through the activation of AMPK.

### Ym155 is more potent inducing cell death than other mitochondrial inhibitors.

**Figure 8:**
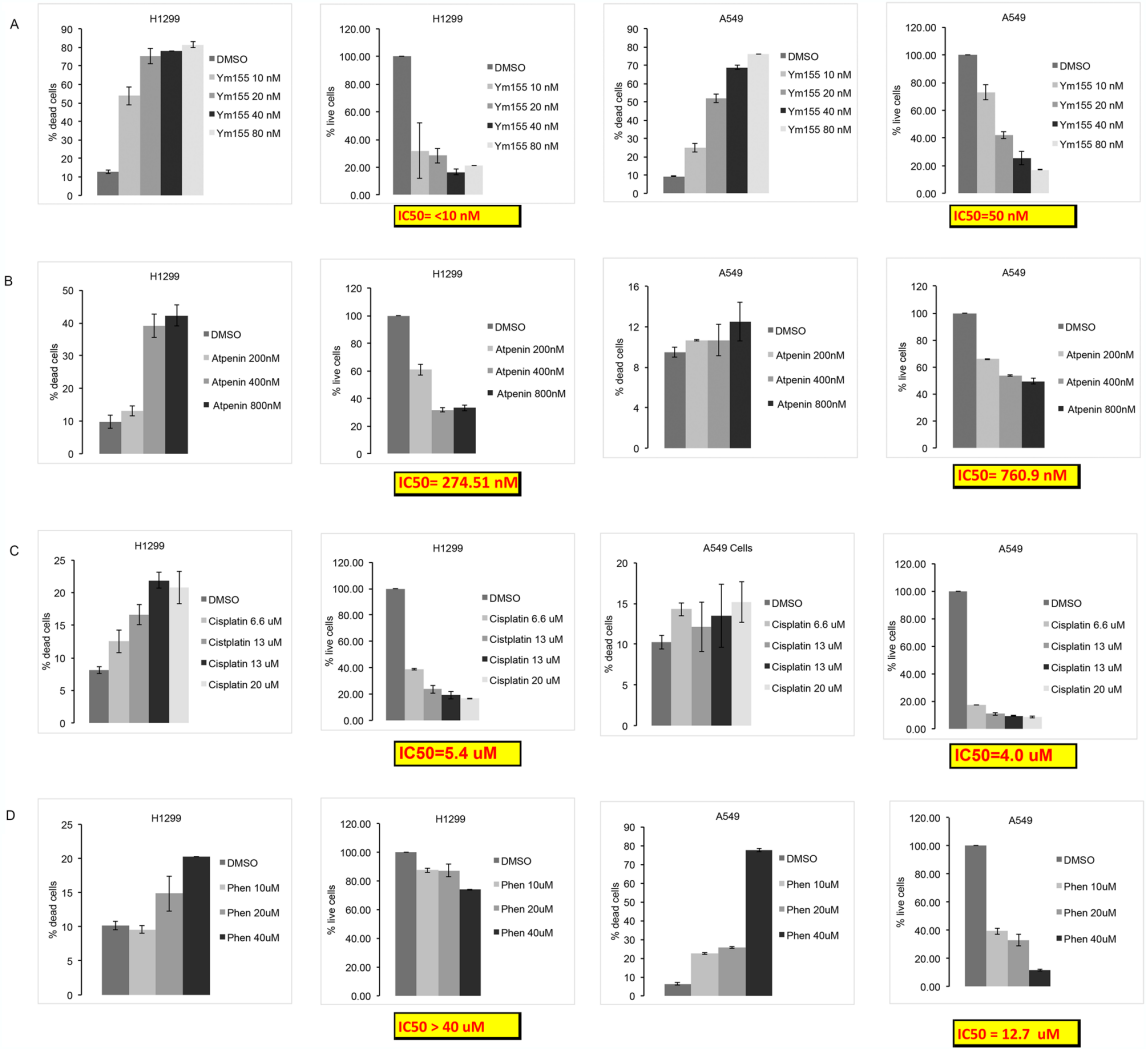
Ym155 is more potent suppressing growth and inducing cell death compared to other mitochondrial inhibitors. H1299 and A549 cells were treated with inhibitor for 48 h. and the percent live and dead cells counted. The data represents the represents mean of 2 experiments treated with (**A**) Ym155, (**B**) Atpenin, (**C**) cisplatin, (**D**) or phenformin.

We compared the potencies of atpenin A5, cisplatin, phenformin, and Ym155 to induce cell death and the concentration that decreases cell number by 50% (IC^50^). Ym155 was significantly more potent inducing cell death and suppressing cell growth of both H1299 and A549 cells (Fig. 8). Ym155 at a dose of 20 nM caused approximately 75% cell death after 48 h and its IC^50^ was < 10 nM and 50 nM in H1299 and A549 cell respectively (Fig. 8A). Atpenin, complex II inhibitor, was the next most potent causing 40% cell death at 400 nM at 48 h in H1299, which was less in A549 cells (Fig. 8B). Even after 5 days of treatment with cisplatin, it required μM concentrations to reach an IC^50^ (Fig. 8C). Consistent with previous a report^32^, phenformin induced more cell death in A549 cells compared to H1299 cells (Fig. 8C). Interestingly, phenformin was the only inhibitor that caused significantly more cell death and growth inhibition in A549 cells in comparison to H1299 cells. Despite the increased potency of phenformin in A549 cells, it is 254 times less potent than Ym155 inhibiting cell growth (Fig. 8D).

## Discussion

We show that Ym155 decreases oxidative phosphorylation, decreases ATP production, decreases mitochondrial membrane potential, increased mitochondrial permeability, decreased TCA cycle intermediates, and activated AMPK in lung cancer cells. These studies support that the mechanism by which Ym155 induces cell death in cancer cells is mediated by disrupting mitochondrial function. The PicoGreen studies suggest that Ym155 localizing to the mitochondria and binds mtDNA, which may be the initiating cause of cell death. Ym155 decreasing oxidative phosphorylation within 2 h demonstrates that it rapidly inhibits the electron transport chain. The TCA cycle was also inhibited by Ym155. Ym155 increased succinate levels and while decreased fumarate and malate intermediates. Complex II is required for the oxidation of succinate to fumarate and is required for the generation of TCA cycle intermediates. These data suggest that Ym155 inhibits the ETC decreasing mitochondrial membrane potential and inhibiting the generation of TCA cycle intermediates.

Targeting the mitochondria is being explored as a means to treat cancer^33^. It has been proposed that the cytotoxic effects of cisplatin are caused by its localization and dysregulation of the mitochondria^30,34^. Atpenin and its analogs demonstrate potent anti-proliferative effects of cancer cells, which are mediated by inhibition of the ETC^35^. Ym155 is significantly more potent suppressing cell growth and inducing cell death of lung cancer cells than the other mitochondrial inhibitors studied. Phenformin and Atpenin only inhibit complex I or complex II respectively of the ECT. Cisplatin does not strongly localize to the mitochondria. Platinum based compounds that that target mtDNA over nuclear DNA have increased cytotoxicity in cancer cells^34^. It is not known why Ym155 is significantly more potent in cell culture than in clinical trials. It is possible that the local tumor microenvironment decreases the ability of Ym155 to localize to the mitochondria. Further studies are needed to answer these questions and determine whether altering drug properties will improve efficacy.

Ym155 inhibits BMP signaling in lung cancer cells at low nanomolar concentrations^15^. We recently showed in both lung cancer cells and *C elegans* that AMPK suppresses BMP signaling^16,17^ Ym155 was also shown to synergize with BMP inhibition to induce AIF caspase-independent cell death that involves the activation of AMPK^16^. Ym155, atpenin, cisplatin, and phenformin all activated AMPK and suppressed BMP signaling in lung cancer cells. Together these data suggest that therapeutics activating AMPK will decrease BMP signaling in cancer cells. The suppression of BMP signaling highlights another potential mechanism by which Ym155 and other cancer therapeutics suppress the growth of cancer cells. BMP ligands are highly over-expressed in non-small cell lung carcinomas compared to normal lung tissue or benign lung tumors^36,37^. Small molecule inhibitors of BMP signaling decrease growth and survival of cancer cells in vitro and in mouse tumor xenografts^38^. Mechanistically BMP signaling cascade regulates several important survival pathways in cancer cells including the activation PI3K^39–41^, increasing expression of anti-apoptotic proteins^42–45^, increase expression of inhibitor of differentiation proteins (Id1-4)^46,47^, and by promoting tumor angiogenesis^48^. Furthermore, BMP inhibition destabilizes microtubules, which enhances lysosome activity and when combined with Ym155 increases lysosome permeability^15^.

## Conclusion

This paper highlights for the first time that Ym155 localizes to the mitochondria promoting mitochondrial dysfunction that induces cell death of lung cancer cells. We demonstrate the Ym155 adaptively increases glycolysis intermediates, while decreasing oxidative phosphorylation and TCA intermediates. Furthermore, we demonstrate that Ym155 and other cancer therapeutics targeting the mitochondria activate AMPK causing a decrease in BMP signaling.

## Methods

### Cell culture and reagents

The A549, H1299 lung cancer cells were cultured in Dulbecco’s modified Eagle’s medium (DMEM, Sigma Aldrich, Saint Louis, MO, USA) with 5% fetal bovine serum. A549-Puro and PicoGreen was obtained from Invitrogen (MA, USA). A549-Puro and A549-LKB1 cell lines were obtained from Prof. Wei Zhou laboratory at Emory University^49^ and were grown with RPMI (Corning) supplemented with 10% FBS and 1% antibiotics. Atpenin A5 (ALX-380–313) was purchased from Enzo Life Sciences and Phenformin hydrochloride (P7045) was obtained from Sigma Aldrich, USA. [U13C6] glucose was purchased from Cambridge Isotope Laboratories.

### Western blot analysis

Western blot analysis was performed as previously reported^36^. In brief, total cellular protein concentration was determined using BCA method then separated by SDS-PAGE and transferred to nitrocellulose (Schleicher and Schuell, Keene, NH). The primary antibodies used were , rabbit monoclonal anti-Smac/Diablo, rabbit monoclonal anti-cytochrome c, rabbit monoclonal anti-activated caspase-3, rabbit monoclonal anti-PARP, (Cell signaling Technology, MA, USA), rabbit monoclonal anti-Id1 (Calbioreagents, San Mateo, CA), rabbit anti-actin, an affinity isolated antigen specific antibody (Sigma, Saint Louis, MO), and rabbit polyclonal anti-GAPDH (Sigma) and mouse monoclonal anti-Spectrin (EMD Millipore, CA, USA). Rabbit monoclonal anti-pAMPKα(T172), rabbit monoclonal anti-AMPK, rabbit monoclonal-pACC(Ser79), rabbit monoclonal-anti-BMPR2 and rabbit monoclonal COX1/MT C01 were purchased from Cell signaling Technology, USA.

### Cell viability

Cells were plated in duplicate into 6-well plates and treated the next day for the designated period of time. Cell counts were determined using the automated cell counter Vi-CELL cell analyzer (Beckman Coulter) as previously reported^15^. Approximately 500 cells per sample were analyzed and trypan blue dye exclusion determined number of dead cells. Cells treated with Ym155, Atpenin, and Phenformin were treated for 48 h and cells treated with cisplatin were treated for 5 days. The cell count experiments were performed at least 2 times.

### Cytosol extraction

Cytosolic protein extraction was performed using Mitochondria/Cytosol fractionation kit as per manufacturer’s instructions (Enzo Life Sciences, NY, USA) and as previously reported^42^. Cell pellets were resuspended in 100 µl of ice-cold Cytosol Extraction Buffer Mix containing dithiothreitol (DTT) and Protease Inhibitors. After a 10 min incubation on ice, cells were homogenized. The homogenates were collected to a fresh 1.5 ml tube and centrifuged at 700 × g for 10 min at 4 °C. The supernatant was collected as the cytosolic fraction and used for further experiments.

### PicoGreen staining

Approximately, 450,000 cells per well were seeded onto microscope coverslips in a 6 well plate for 24 h. A549 cells were treated with increasing concentrations of Ym155 for 1 h. After treatment, cells were stained with PicoGreen solution (Molecular Probes Inc.) for 1 h at 37 °C in 5% carbon dioxide (CO2). 3 µl/ml stock PicoGreen solution was added into 1 ml of cell culture media. Cells were washed with PBS and mounted with ProLong Diamond Antifade Mountant (Invitrogen), dried overnight and examined under a fluorescence microscope (Nikon eclipse TE300). The number of mitochondria staining with PicoGreen was counted in each cell using ImageJ (NIH) and depicted as the average number of nucleoids per cell. Approximately 50 cells were counted in each condition. The experiment was replicated two times.

### Adenosine triphosphate (ATP) assay

Trichloroacetic acid (TCA) method for ATP extraction was performed according to the manufacturer’s protocol (Enliten®ATP Assay System, Promega). Briefly, 600,000 cells/ well cells were plated and grown overnight at 37 °C in 5% carbon dioxide (CO2). Cells were treated in duplicate and after treatment, cell pellets were collected by trypsinization, washed with PBS and transferred into fresh 1.5 ml tubes. The cell suspension was centrifuged at 1500 rpm for 5 min. 150 µl of ice cold 1% TCA was added into the cell pellet and mixed for 40 s. The mixture was centrifuged at 13,000 × g for 5 min at 4 °C. The ATP extract was immediately 100-fold diluted in 1 M tris–acetate buffer (pH 7.75). Intracellular ATP was measured by Enliten®ATP Assay System using a luminometer (Tecan, Infinite M200Pro). The experiment was replicated three times in our laboratory.

### Immunofluorescence staining

Immunofluorescence was performed as previously reported^15^. Cells were seeded for 24 h onto microscope cover glasses in a 6-well plate then treated. Cells were fixed with 4% formaldehyde for 20 min at room temperature and then permeabilized with 0.5% triton-X for 20 min at room temperature. Cells were blocked with CAS-block for 1 h. The cells were stained with rabbit monoclonal anti-TUFM antibody (abcam, USA) at 1:500 dilution for 1 h. Then the cells were washed with PBS and stained with antirabbit Alexa Flour 568 conjugated secondary antibody (Invitrogen, USA) for 1 h. After 1 h. cells were washed with PBS once and then with distilled water. The cover glasses were mounted with ProLong Diamond Antifade Mountant (Invitrogen), dried overnight and examined under a fluorescence microscope (Nikon eclipse TE300).

### TMRM staining

For TMRM staining, 400,000 cells/ well were seeded in a 6-well plate and grown overnight. Next day, cells were treated with DMSO, 10 nM Ym 155 and 20 nM Ym155 for three hours. After treatment, cells were washed with PBS and stained with TMRM (Thermo Scientific# M20036) at a concentration of 20 nM for 30 min at 37 degree in 5% CO2. Cells were then washed with PBS and the images were taken using 20X objective in a fluorescence microscope (Nikon eclipse TE300). The fluorescence intensity was quantified with ImageJ (NIH) software. ImageJ (Fig. 2F) is available for free software provided to researchers provided by the NIH at https://imagej.nih.gov/ij/download.html.

### Mitochondrial respiration

Oxygen consumption rate (OCR) and ATP production in H1299 cells after the Ym155 treatment, was measured using a Seahorse Biosciences extracellular flux analyzer (XF24) as described previously^50,51^. Briefly, H1299 cells were seeded at 2.5 × 104 cells per well in the XF24 plates and incubated at 37 °C and 5% CO2 overnight. Next day, cells were treated with vehicle DMSO, Ym 155 10 nM, 20 nM, and 40 nM in RPMI medium for 2 h and basal OCR as well as ATP levels were measured. The results were analyzed using Wave software. Wave software (Fig. 3) is available for free for Seahorse data analysis at https://www.agilent.com/en/product/cell-analysis/real-time-cell-metabolic-analysis/xf-software/seahorse-wave-desktop-software-740897.

### Metabolomic analysis by LC–MS

To assay the total level of metabolite pools, H1299 cells were seeded at 1.6 × 10^6^ cells per plate in 60 mm dishes and incubated overnight. Cells were treated with DMSO, Ym155 10 nM and Ym155 40 mM in RPMI medium for 3 h. The media was aspirated, then quickly overlaid with a 40:40:20 buffer of methanol: acetonitrile: water and 0.5% formic acid. The plates were incubated on ice for 10 min, then 15% ammonium bicarbonate was added to neutralize the acetic acid. The cells were scraped from the plates using a cell lifter, transferred to 1.5 mL microcentrifuge tubes on ice, and centrifuged for 10 min at 13,000 rpm at 4 °C. The supernatants were transferred to LC–MS autosampler vials (on ice) and sent for LC–MS analysis.

To analyze the effect of Ym155 on metabolic pathways by tracing glucose metabolism, H1299 cells were seeded at 1.6 × 106 cells per plate in 60 mm dishes and incubated overnight. Next day, the media was aspirated and cells were supplemented with fresh DMEM containing 1000 mg/L [U13C6] glucose (Chembribge Isotope laboratories # CLM-1396). Then the cells were treated with DMSO, Ym155 10 nM and Ym155 40 mM for 3 h. Cellular metabolites were collected as described above and sent for LC–MS analysis.

LC–MS analysis of the cellular metabolites was performed on the Q Exactive PLUS hybrid quadrupole-orbitrap mass spectrometer (Thermo Scientific) coupled to hydrophilic interaction chromatography (HILIC) as previously reported ^50,51^. The LC separation was performed on UltiMate 3000 UHPLC system with an XBridge BEH Amide column (150 mm × 2.1 mm, 2.5 μM particle size, Waters, Milford, MA) with the corresponding XP VanGuard Cartridge. The liquid chromatography used a gradient of solvent A (95%:5% H2O: acetonitrile with 20 mM ammonium acetate, 20 mM ammonium hydroxide, pH 9.4), and solvent B (20%:80% H2O: acetonitrile with 20 mM ammonium acetate, 20 mM ammonium hydroxide, pH 9.4). The gradient was 0 min, 100% B; 3 min, 100% B; 3.2 min, 90% B; 6.2 min, 90% B; 6.5 min, 80% B; 10.5 min, 80% B; 10.7 min, 70% B; 13.5 min, 70% B; 13.7 min, 45% B; 16 min, 45% B; 16.5 min, 100% B. The flow rate was 300 μl/min. Injection volume was 5 μL and column temperature 25 °C. The MS scans were in negative ion mode with a resolution of 70,000 at m/z 200. The automatic gain control (AGC) target was 3 × 10^6^ and the scan range was 75 − 1000. Metabolite features were extracted in MAVEN^52^. The metabolite annotations are made by matching the accurate mass and retention time to our in-house metabolite database, which covers > 400 polar metabolites.

### Determination of electrostatic charge distribution

The comparison of the electrostatic charge distribution of Ym155 and ethidium bromide was determined using Molecular Operating Environment (MOE) (Fig. 1B). Molecular Operating Environment (MOE) software (v.2019.0102) licensed from Chemical Computing Group's (https://www.chemcomp.com/index.htm) via Rutgers University Office of Advanced Research Computing (https://oarc.rutgers.edu/).

### Statistical analysis

The mean of the control group was compared to the mean of each treated group using a paired student t-test assuming unequal variances. Differences with *p* values < 0.05 were considered statistically significant.

## Supplementary Information

Supplementary Information 1.

Supplementary Information 2.

Supplementary Information 3.

## Data Availability

The datasets obtained and analyzed for this study will be made available from the corresponding author in a reasonable request.
